# Secretion and N-Linked Glycosylation Are Required for Prostatic Acid Phosphatase Catalytic and Antinociceptive Activity

**DOI:** 10.1371/journal.pone.0032741

**Published:** 2012-02-28

**Authors:** Julie K. Hurt, Brendan J. Fitzpatrick, Jacqueline Norris-Drouin, Mark J. Zylka

**Affiliations:** 1 Department of Cell and Molecular Physiology, Neuroscience Center, University of North Carolina, Chapel Hill, North Carolina, United States of America; 2 Center for Integrative Chemical Biology and Drug Discovery, University of North Carolina, Chapel Hill, North Carolina, United States of America; German Institute of Human Nutrition Potsdam-Rehbruecke, Germany

## Abstract

Secretory human prostatic acid phosphatase (hPAP) is glycosylated at three asparagine residues (N62, N188, N301) and has potent antinociceptive effects when administered to mice. Currently, it is unknown if these N-linked residues are required for hPAP protein stability and activity *in vitro* or in animal models of chronic pain. Here, we expressed wild-type hPAP and a series of *Asn* to *Gln* point mutations in the yeast *Pichia pastoris* X33 then analyzed protein levels and enzyme activity in cell lysates and in conditioned media. *Pichia* secreted wild-type recombinant (r)-hPAP into the media (6–7 mg protein/L). This protein was as active as native hPAP in biochemical assays and in mouse models of inflammatory pain and neuropathic pain. In contrast, the N62Q and N188Q single mutants and the N62Q, N188Q double mutant were expressed at lower levels and were less active than wild-type r-hPAP. The purified N62Q, N188Q double mutant protein was also 1.9 fold less active *in vivo*. The N301Q mutant was not expressed, suggesting a critical role for this residue in protein stability. To explicitly test the importance of secretion, a construct lacking the signal peptide of hPAP was expressed in *Pichia* and assayed. This “cellular” construct was not expressed at levels detectable by western blotting. Taken together, these data indicate that secretion and post-translational carbohydrate modifications are required for PAP protein stability and catalytic activity. Moreover, our findings indicate that recombinant hPAP can be produced in *Pichia*—a yeast strain that is used to generate biologics for therapeutic purposes.

## Introduction

*Prostatic acid phosphatase* (*Pap*, also known as *Acpp*) is a single gene that encodes two extracellularly active enzymes: 1. secretory (S-) and 2. transmembrane (TM-) PAP [1]. S-PAP is expressed in prostate epithelial cells and has long been used as a prostate cancer biomarker [2]. S-PAP was thought to be prostate specific; however, recent studies revealed that the splice variant (TM-PAP) was expressed in additional tissues, including salivary gland, lung, kidney, skeletal muscle and nociceptive (pain-sensing) dorsal root ganglia neurons [1], [3]. While PAP was classically considered to be a non-specific phosphomonoesterase (E.C. 3.1.3.2) [4], *in vivo* PAP functions as an ectonucleotidase that hydrolyzes extracellular adenosine 5′-monophosphate (AMP) to adenosine [3]. Deletion of PAP reduces extracellular AMP hydrolysis in nociceptive neurons and in the dorsal spinal cord [5]. Moreover, S-PAP (injected intrathecally) has long-lasting (three-day) antinociceptive effects in mouse models of inflammatory pain and neuropathic pain and these antinociceptive effects are entirely adenosine A_1_ receptor (A_1_R) dependent [3], [6]. In addition, S-PAP has enduring (>7 days) A_1_R-dependent antinociceptive effects if injected intrathecally before nerve injury or inflammation [7]. These findings suggest a recombinant version of human S-PAP could be used as a treatment for chronic pain or for preemptive analgesia [8].

The structure and active site of S-PAP has been extensively characterized from various species [9]. Mammalian S-PAP exists primarily as a homodimer made up of two 50 kDa subunits [10]. Mutations that disrupt dimerization eliminate catalytic activity [11]. S-PAP is present at high concentrations in human semen, which has facilitated purification and crystallization of the native protein. Each subunit of the native human enzyme is post-translationally modified with N-linked carbohydrate residues at three asparagine residues (N62, N188, N301) [12]. The crystal structure of recombinant rat S-PAP, produced in insect cells, was also solved and contains N-linked carbohydrates at two of these three conserved asparagine residues (N62 and N301 but not N188) [13].

PAP is classified as a histidine phosphatase because the catalytic residue in the enzyme-substrate intermediate is H12 [14]. Site-directed mutagenesis of H12 and amino acids in the enzyme active site (R11, R15, H257, and D258) revealed important roles for these residues in catalysis [11], [15]. In addition, X-ray crystallographic studies revealed interactions between active site amino acids and a known PAP inhibitor, L-(+)-tartrate [16], [17], [18].

Though the breadth of mutational analysis led to significant understanding of residues important for catalysis, it is currently unknown if secretion or N-linked glycosylation is required for the stability and activity of PAP. In the present study, we expressed recombinant human S-PAP (r-hPAP) in the methylotrophic yeast species, *Pichia pastoris* X33. Our goals were to determine if biologically active r-hPAP enzyme could be produced in *Pichia*—a eukaryotic species that is used to generate recombinant proteins for clinical applications [19], [20]. In addition, we sought to determine if each of the known N-linked carbohydrate residues were important for hPAP stability and biological activity.

## Results

### *In vitro* activity of recombinant hPAP from *Pichia pastoris* X33

**Figure 1:**
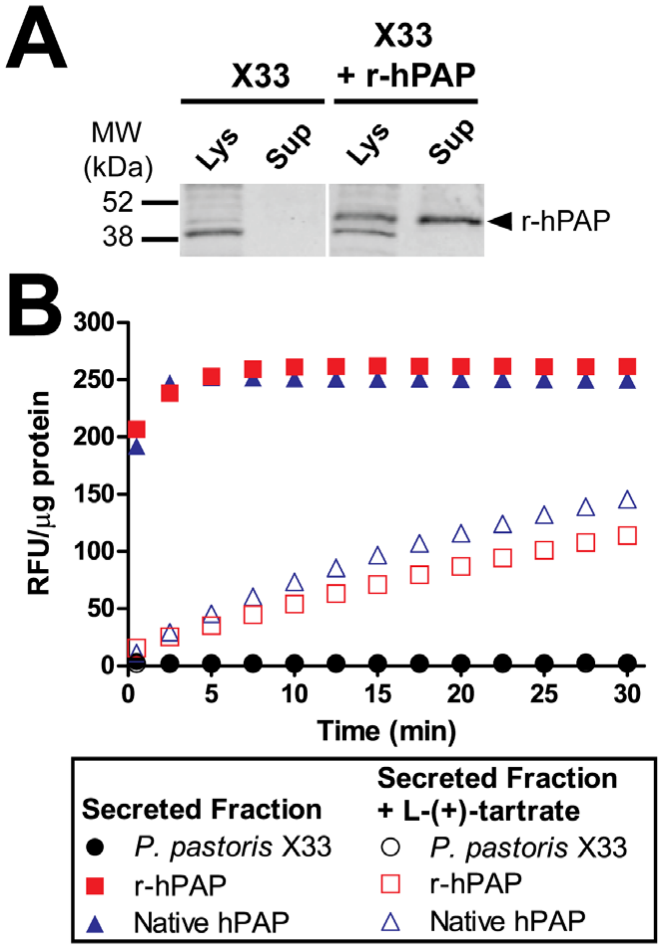
*Pichia*-derived r-hPAP is secreted and catalytically active. (A) Western blot of crude cell lysates (Lys) and supernatants (Sup) from *P. pastoris* X33 untransformed controls and from *P. pastoris* X33 expressing r-hPAP. Blot probed with anti-hPAP antiserum. (B) DiFMUP fluorometric enzyme assay with concentrated supernatants (secreted fractions) in comparison to native hPAP from human semen. 0.625 µg total protein used per reaction. Data are plotted as an average of duplicate trials ± standard deviation (SD).

To determine if recombinant human S-PAP (r-hPAP) could be produced in *Pichia*, we stably integrated a wild-type version of S-PAP into the *Pichia* genome under the control of the methanol-inducible *AOX1* promoter. Fourty-eight hours after induction with 1% methanol, conditioned medium and crude cell pellets were collected for western blotting and enzyme assays. On denaturing western blots, we observed a single 50 kDa immunoreactive band in conditioned media (the secreted fraction) from r-hPAP transformants but not in media from untransformed *P. pastoris* X33 controls (Figure 1A). This 50 kDa band corresponded to the known molecular weight of native S-PAP [21], and indicated that r-hPAP was secreted into the medium by *Pichia*. r-hPAP was also detected in the lysate (intracellular fraction) at the 48 hour time point (Figure 1A), reflective of full-length protein within secretory organelles (signal peptide-bearing proteins are not trafficked to the cytoplasm). Lysates from untransformed X33 cells and r-hPAP transformants also contained a cross-reactive (non-PAP specific) 38 kDa band.

The secreted fraction from r-hPAP transformants contained L-(+)-tartrate-sensitive phosphatase activity that was equivalent in activity to native, semen-derived hPAP (Figure 1B). In contrast, neither the intracellular nor secreted fraction from untransformed *P. pastoris* X33 cells had measureable phosphatase activity (Figure 1B, Table 1). Taken together, these data indicate that r-hPAP is secreted from *Pichia* in a catalytically active form.

**Table 1:** Phosphatase activity of r-hPAP integrants.

	Cell Lysates		Secreted Fractions		
*Pichia* Integrant	RFU/µg protein#(± SD)	Rel. Activity	RFU/µg protein#(± SD)	Rel. Activity	AUC*(± SEM)
*P. pastoris* X33	1.0 (0.03)	<0.01	1.8 (0.05)	<0.01	**-**
r-hPAP	12.2 (0.28)	1	109.5 (1.44)	1	282 (21)
r-hPAP (N62Q)	7.8 (0.29)	0.64	67.0 (2.78)	0.61	n.t.
r-hPAP (N188Q)	3.3 (0.16)	0.27	26.2 (0.45)	0.24	n.t.
r-hPAP (N301Q)	1.1 (0.01)	<0.01	2.0 (0.12)	<0.01	n.t.
r-hPAP (N62Q, N188Q)	2.5 (0.05)	0.20	17.6 (1.09)	0.16	149 (25)
r-hPAP (N62Q, N301Q)	0.8 (0.03)	<0.01	1.8 (0.05)	<0.01	n.t.
r-hPAP (N188Q, N301Q)	1.0 (0.03)	<0.01	1.9 (0.01)	<0.01	n.t.
r-hPAP (N62Q, N188Q, N301Q)	1.0 (0.06)	<0.01	1.7 (0.11)	<0.01	n.t.
r-hPAP (-SP)	0.9 (0.02)	<0.01	2.0 (0.02)	<0.01	n.t.

# RFU at 10 min DiFMUP assay timepoint, normalized to total protein.

* AUC of antinociceptive effects shown in Figure 5.

n.t., not tested.

### N-linked glycosylation of recombinant hPAP in *Pichia*

**Figure 2:**
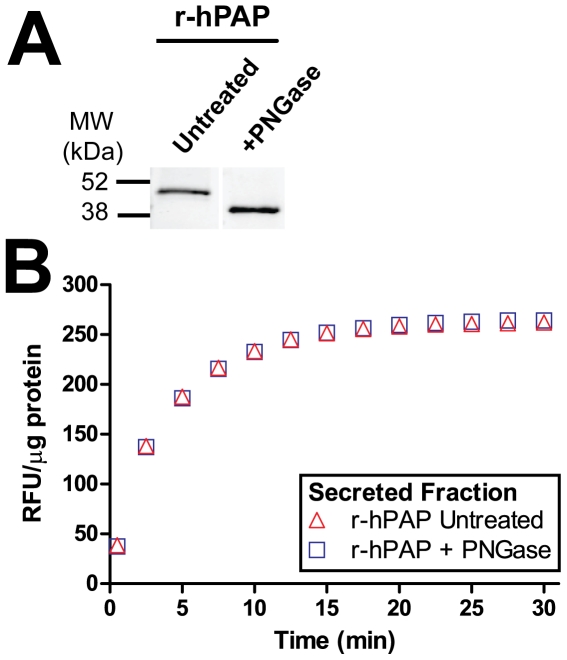
r-hPAP is glycosylated when expressed in *Pichia*. (A) Western blot of concentrated r-hPAP secreted fraction after incubation at 37°C for 24 h with or without 1000 U PNGase. Blot probed with anti-hPAP antiserum. (B) DiFMUP fluorometric enzyme assay using equal amounts of untreated and PNGase-treated r-hPAP. Data are plotted as an average of duplicate trials ± SD.

To determine if this secreted *Pichia*-derived protein was glycosylated, we treated cell supernatants containing r-hPAP with the enzyme N-glycosidase F (PNGase F) for 24 hr. Following treatment, the molecular weight of r-hPAP was reduced to ∼40 kDa (Figure 2A). The phosphatase activity of PNGase-treated r-hPAP was equivalent to untreated r-hPAP (Figure 2B), indicating removal of solvent accessible carbohydrates from the mature, fully processed protein does not affect activity.

### Critical role for N-linked residues in hPAP expression

Native hPAP is glycosylated at three asparagine residues (N62, N188, N301) [22]. To determine if these N-linked residues were important for protein expression, enzyme activity or secretion, we generated single mutants of each residue, three double mutants, and one triple mutant (see methods). We confirmed that each r-hPAP mutant was stably integrated into the *AOX1* locus by colony PCR (Figure S1).

**Figure 3:**
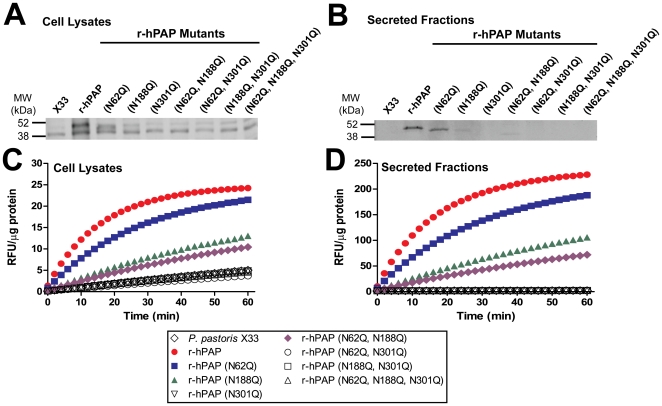
Expression and activity of N-linked glycosylation mutants. Western blots of (A) crude cell lysates and (B) crude secreted fractions from the indicated *P. pastoris* X33 integrants. Blots probed with anti-hPAP antiserum. Equivalent amounts of total protein were loaded in each lane. (C, D) DiFMUP fluorometric enzyme assays of (C) crude cell lysates and (D) crude secreted fractions. Data are plotted as an average of duplicate trials ± SD.

After inducing protein expression, the r-hPAP (N62Q) mutant ran at a lower molecular weight than wild-type r-hPAP (Figure 3A, B), likely due to the loss of a single high-mannose glycan at residue 62 (approximately 2 kDa) [22]. The N62 mutant was secreted and expressed at similar levels as wild-type r-hPAP (Figure 3B), although this N62 mutant was significantly less active in comparison to wild-type r-hPAP (61% relative activity) (Figure 3C, D, Table 1). The r-hPAP (N188Q) and (N62, N188Q) double mutant were also secreted (Figure 3B), although these mutants were expressed at very low levels and had correspondingly low levels of activity (24% and 16% in comparison to r-hPAP, respectively) (Figure 3C, D, Table 1). In addition, all of these r-hPAP mutants were inhibited by saturating concentrations of L-(+)-tartrate (Figure S2A–B). Strikingly, none of the hPAP mutants containing the N301Q mutation were expressed in *Pichia* (Figure 3A and 3B), an observation that was confirmed with enzyme assays (Figure 3C,D, Table 1), suggesting an essential role for this N-linked residue in protein expression and stability.

### hPAP secretion is critical for activity *in vitro*

**Figure 4:**
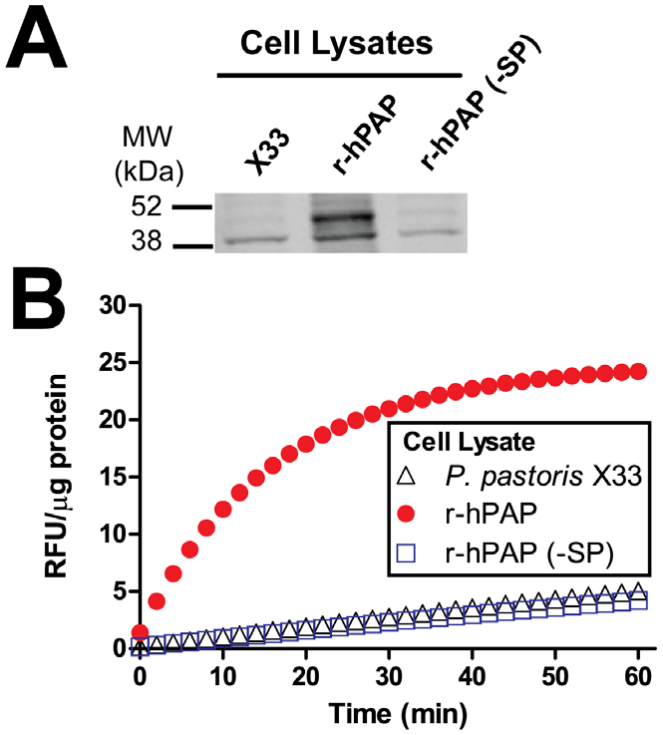
Expression and activity of r-hPAP lacking signal peptide(-SP). (A) Western blot of crude cell lysates from untransformed X33 cells and transformants expressing hPAP with (r-hPAP) or without (-SP) a signal peptide. Blot probed with anti-hPAP antiserum. Equivalent amounts of total protein were loaded in each lane. (B) DiFMUP fluorometric enzyme assay with the indicated crude cell lysates. Data are plotted as an average of duplicate trials ± SD.

Since proteins are glycosylated while in transit through the secretory pathway, and mutation of a single N-linked residue eliminated hPAP expression, we hypothesized that hPAP expression would also be dependent on secretion. To test this hypothesis, we generated a version of r-hPAP that was otherwise identical to wild-type r-hPAP except for removal of the α-factor signal peptide (-SP). This r-hPAP(-SP) clone was stably integrated into the *Pichia AOX1* locus (Figure S1). However, this mutant was not detectably expressed or active (Figure 4A, B), suggesting that hPAP must transit through the secretory pathway for proper expression and activity.

### Recombinant hPAP has antinociceptive activity

**Figure 5:**
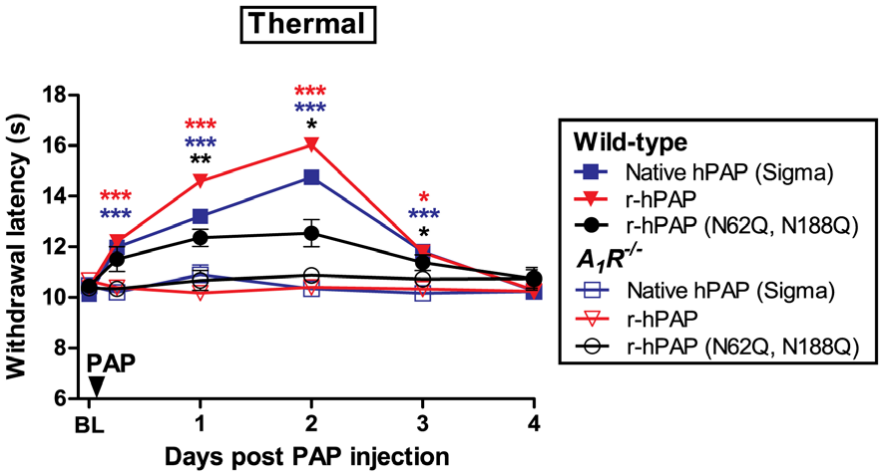
r-hPAP has antinociceptive properties *in vivo*. Antinociceptive properties of native hPAP, r-hPAP and r-hPAP (N62Q, N188Q) injected intrathecally into wild-type (n = 10) and *A_1_R* ^−/−^ mice (n = 10). Equivalent unit amounts (250 mU/mouse) of native hPAP and r-hPAP were injected. Equivalent protein amounts (0.21 mg/mL) of r-hPAP and r-hPAP (N62Q, N188Q) were injected. Paired t-tests were used to compare responses at each time point to baseline (BL). *p<0.05, **p<0.005, ***p<0.0005. Data are plotted as means ± standard error of the mean (SEM).

**Figure 6:**
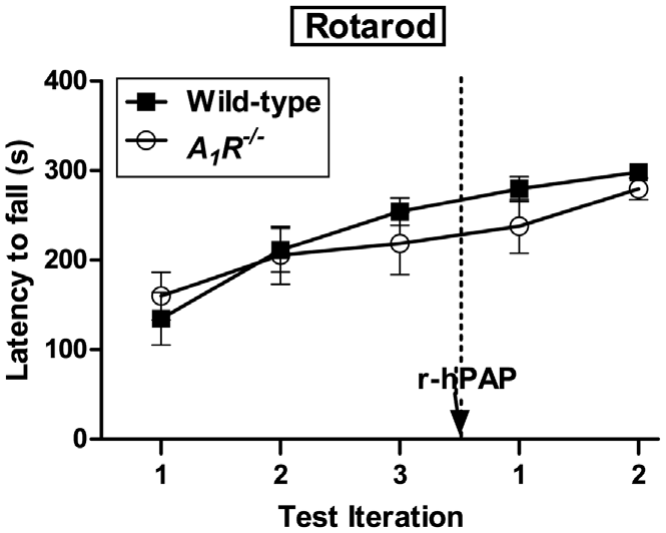
r-hPAP does not affect balance or motor function in mice. Rotarod tests with wild-type (n = 10) and A_1_R^−/−^ mice (n = 10) 24 h before (3 iterations, separated by 40 s) and 24 h after (2 test iterations, separated by 40 s) intrathecal injection of r-hPAP (250 mU/mouse). No significant differences between genotypes or treatment. Data are plotted as means ± SEM. The same mice shown were also tested for thermal sensitivity, to confirm that hPAP injections were successful (as evidenced by a significant thermal antinociceptive effect in wild-type mice injected with hPAP, data not shown).

Given the potential use of r-hPAP as a therapeutic in humans, we next sought to determine if purified r-hPAP protein had antinociceptive activity in preclinical models of pain. To test this, we purified r-hPAP to homogeneity (see methods; typical yield 0.5 mg/L supernatant). We then measured noxious thermal sensitivity before and after injecting pure r-hPAP (250 mU) intrathecally into wild-type and *A_1_R^−/−^* mice. In parallel, native hPAP (250 mU) and an equivalent amount of purified r-hPAP (N62Q, N188Q) were injected into additional groups of WT and *A_1_R^−/−^* mice. We observed long-lasting antinociceptive activity (three days) in WT mice following a single injection of each protein (Figure 5). Area under the curve (AUC) calculations revealed comparable antinociceptive activity with native hPAP and purified r-hPAP in wild-type mice (232±29.7 and 282±20.7, respectively). The r-hPAP (N62Q, N188Q) protein was significantly less effective (AUC 149±24.5), approximately 53% of the AUC determined for r-hPAP (Figure 5 and Table 1), consistent with reduced activity of this protein *in vitro* (Table 1). None of the proteins tested had any effect in *A_1_R^−/−^* mice (Figure 5), consistent with our previous work showing that the antinociceptive effects of PAP were entirely dependent on adenosine A_1_ receptor activation [3], [6]. These antinociceptive effects were not due to reduced motor function, as r-hPAP did not affect performance of wild-type or *A_1_R^−/−^* mice on the rotarod test (Figure 6), a quantitative measure of balance and mobility.

**Figure 7:**
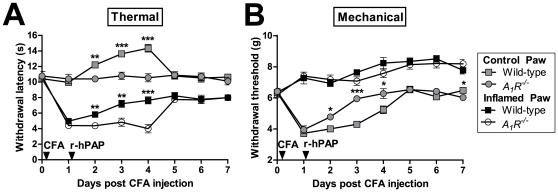
Antinociceptive effects of r-hPAP in chronic inflammatory pain model. (A, B) CFA was injected into one hindpaw (CFA-arrow) of wild-type (n = 10) and *A_1_R^−/−^* mice (n = 10). r-hPAP (250 mU) was intrathecally injected 1 day later (r-hPAP-arrow). Inflamed and non-inflamed (control) hindpaws were tested for (A) thermal and (B) mechanical sensitivity. Data are plotted as means ± SEM. Paired t-tests were used to compare responses at each time point between genotypes, same paw comparisons. *p<0.05, **p<0.005, ***p<0.0005.

We next measured noxious thermal and mechanical sensitivity before and after inflaming one hindpaw of wild-type and *A_1_R^−/−^* mice with Complete Freund's adjuvant (CFA, Figure 7). In this inflammatory pain model, we found that r-hPAP (250 mU, single intrathecal injection) had long-lasting (three day) thermal anti-hyperalgesic and mechanical anti-allodynic effects in WT mice but not *A_1_R^−/−^* mice (inflamed paw, Figure 7A, B).

**Figure 8:**
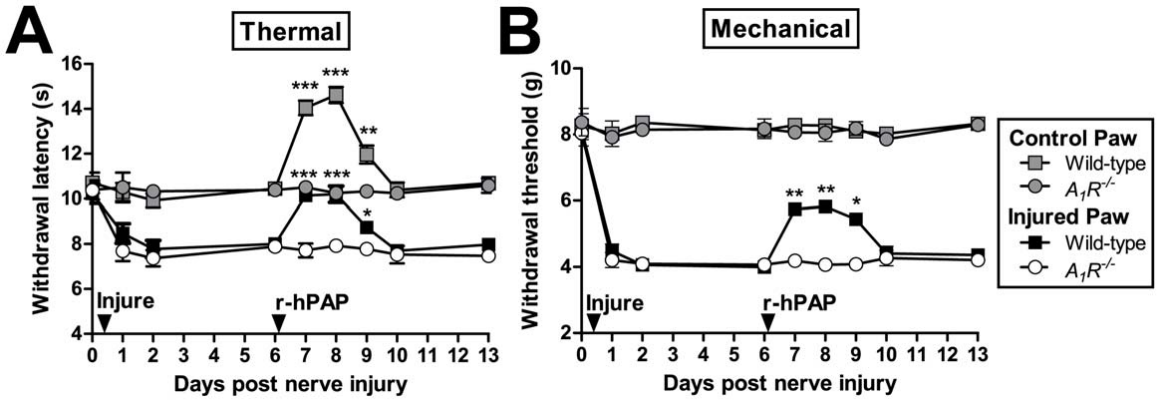
Antinociceptive effects of r-hPAP in neuropathic pain model. (A, B) The sural and common peroneal branches of the sciatic nerve were ligated and then transected (injure-arrow) in wild-type (n = 10) and *A_1_R^−/−^* mice (n = 10). Six days later, r-hPAP (250 mU) was injected intrathecally. Injured and non-injured (control) hindpaws were tested for (A) thermal and (B) mechanical sensitivity. Data are plotted as means ± SEM. Paired t-tests were used to compare responses at each time point between genotypes, same paw comparisons. *p<0.05, **p<0.005, ***p<0.0005.

In a separate group of wild-type and *A_1_R^−/−^* mice, we surgically cut two of the three branches of the sciatic nerve to model neuropathic pain (Figure 8). Six days later, mice were injected intrathecally with r-hPAP (250 mU). We found that r-hPAP had long-lasting (three day) thermal anti-hyperalgesic and mechanical anti-allodynic effects WT mice but not *A_1_R^−/−^* mice (injured paw, Figure 8A, B). Taken together, these data reveal that r-hPAP, like native hPAP from semen, has long-lasting antinociceptive effects in two preclinical models of chronic pain [3].

## Discussion

### PAP catalytic activity requires N-linked glycosylation sites

**Figure 9:**
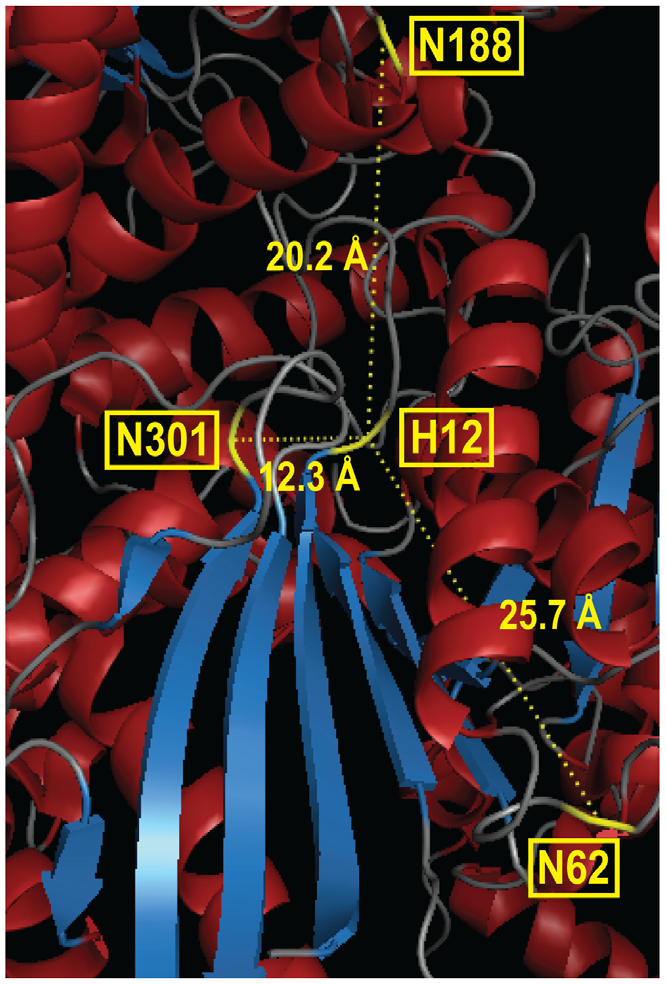
Location of N-linked asparagines residues relative to the active site of hPAP. The x-ray crystallographic structure depicts the essential active site residue H12 and the three N-linked residues (highlighted in yellow) in one subunit of native hPAP. Distances were calculated in PyMOL between the alpha carbon of each amino acid. Structure coordinates from PDB #1ND6 [18].

Native hPAP is post-translationally modified with N-linked glycans at three solvent exposed asparagine residues: N62, N188 and N301 [12], [18]. To understand the role each N-linked residue plays in protein expression and activity, we generated a series of r-hPAP N-glycosylation point mutants and stably introduced these clones into a defined region of the *Pichia* genome. Three of these mutants [r-hPAP (N62Q), r-hPAP (N188Q) and r-hPAP (N62Q, N188Q)] were expressed and active (albeit less active than wild-type r-hPAP) in the cellular fraction and secreted fraction, suggesting key elements of the tertiary and quaternary r-hPAP structure were conserved in these mutants. Of these N-linked residues, N62 is farthest from H12 in the active site of hPAP, N188 is at an intermediate distance, while N301 is closest to the active site (25.7 Å, 20.2 Å and 12.3 Å between the alpha carbons of each residue, respectively) (Figure 9). Correspondingly, the N62Q mutant had the smallest effect on protein expression and catalytic activity when mutated (61% activity relative to r-hPAP) (Figure 3, Table 1). Mutation of N188 resulted in a greater loss of activity (24% activity relative to r-hPAP) (Table 1), while the hPAP (N62Q, N188Q) double mutant activity was further reduced (16% activity relative to r-hPAP) (Table 1). These data thus suggest that mutation of N-linked residues closer to the active site have a greater effect on protein activity and stability.

Indeed, no stable protein product was observed in the cellular or secreted fraction in any r-hPAP integrant bearing the N301Q mutation. N301 is closely oriented with several polar residues in the enzyme active site, namely two arginine residues (R11 and R15) and the catalytic residue, H12 (Figure 9) [18]. The glycoside moiety of N301 may thus form hydrogen-bonds with amino acids in or near the active site to direct proper protein folding. In contrast, the distances between these N-linked residues and H12 of the neighboring subunit in the homodimer is far greater (26.9 Å for N62, 43.8 Å for N188, and 38.9 Å for N301). This suggests the N-linked glycans likely contribute to intrasubunit folding and stability as opposed to intersubunit stability.

Our findings could explain why a previous attempt to express active recombinant PAP in *E. coli* proved unsuccessful [15], as *E. coli* do not glycosylate proteins on asparagine residues [23]. Post-translational modifications like N-linked glycosylation help direct protein folding and protein secretion in the endoplasmic reticulum [24]. Without these modifications, misfolded proteins are not carried through the secretory pathway, but are instead rapidly degraded. This likely explains why we saw reduced amounts of PAP or no PAP in our various N-linked mutants. Likewise, acid phosphatase from *S. cerevisiae* aggregated in the endoplasmic reticulum and was not secreted when N-linked glycosylation was impaired [25], [26].

In contrast, when carbohydrates were removed from the fully-folded and functional r-hPAP protein, we saw no loss of stability or activity *in vitro* (Figure 2B). This is consistent with a previous report showing that removal of N-linked glycans from fully-folded native PAP did not diminish catalytic activity, although stability was reduced at high pH [21].

Our findings also have implications for studies describing a “cellular” version of PAP that reportedly dephosphorylates amino acid residues in the cytoplasm [27], [28], [29]. Given that proteins are glycosylated when in transit through the secretory pathway, that PAP requires N-linked glycosylation for activity, and that a “cellular/cytoplasmic” version of PAP lacking a signal peptide was not expressed or active (Figure 4), our data reveal that it is not possible for PAP, when expressed by cells, to be active in the cytoplasm. Moreover, despite extensive sequencing of transcripts from the mouse and human genome, including numerous transcripts encoding S- and TM-PAP in dbEST, there are no transcripts encoding a cellular isoform of PAP (i.e., a transcript lacking a signal peptide). As a result, any study suggesting that PAP directly dephosphorylates residues within the cytoplasm must be reinterpreted as indirect [27], [28], [29].

### Recombinant S-PAP is as effective as native S-PAP in preclinical models of chronic pain

We previously found that native hPAP, purified from human semen, and recombinant mouse PAP, purified using a baculovirus expression system, had long-lasting A_1_R-dependent antinociceptive effects in mouse models of inflammatory pain and neuropathic pain [3], [6], [7]. While the baculovirus expression system is ideal for generating protein for experimental purposes, this system has only recently been used to make recombinant protein for clinical applications [30], [31]. In contrast, *Pichia pastoris* is a well characterized eukaryotic expression system and has been used to make pharmaceutical-grade recombinant proteins for diverse applications [19], [20]. Here, we expressed r-hPAP under aerobic conditions in *Pichia* and obtained protein yields (crude yield 6–7 mg hPAP/L culture) that were significantly higher than when r-hPAP was produced in a different yeast species, *Saccharomyces cerevisiae* (crude yield 0.5 mg hPAP/L culture) [15]. Protein yields are typically much higher in *Pichia* under fermentation conditions (grams of protein/L) [20], so it may be possible to further increase yields of r-hPAP under fermentation conditions.

We found that *Pichia*-derived r-hPAP was as effective as native hPAP *in vivo* and had A_1_R-dependent antinociceptive effects in preclinical models of inflammatory pain and neuropathic pain. Purified r-hPAP (N62Q, N188Q) also had antinociceptive effects that lasted for three days, although the magnitude of this effect was reduced (53% relative to purified r-hPAP) (Table 1). Note that the same amount of total protein was used in these experiments although the enzyme activity differed. Remarkably, when combined with our previous work showing that heat denaturation eliminates PAP catalytic activity and antinociceptive activity [3], there appears to be a direct relationship between catalytic activity and the magnitude of antinociceptive activity. It should thus be possible to biochemically tune the A_1_R-dependent antinociceptive effects of PAP by introducing mutations that increase or decrease catalytic activity. Ultimately, our study highlights a critical role for post-translational modifications in regulating catalytic activity and provides a way to generate large amounts of recombinant hPAP for future clinical trials.

## Materials and Methods

### hPAP constructs

Secretory hPAP was amplified without its signal peptide (nt. 187–1248, GenBank accession # NM_001099.4) from human placenta cDNA with Phusion DNA polymerase (New England Biolabs). The amplified product was subcloned into the *Pichia pastoris* expression vector pPICZαA (Invitrogen) in-frame with the *AOX1* promoter and α-factor signal peptide. We refer to this clone as recombinant wild-type hPAP (r-hPAP) because the α-factor yeast signal peptide is removed upon secretion, generating a protein that is identical in sequence to wild-type S-PAP. r-hPAP(-SP), a “cytoplasmic/cellular” version of PAP that lacks a signal peptide, contains a 5′ methionine codon (*atg*) in-frame with hPAP (nt. 187–1248, GenBank accession # NM_001099.4) subcloned into pPICZB. All constructs were confirmed by DNA sequencing.

### Site-directed mutagenesis

QuikChange® multi site-directed mutagenesis (Stratagene) was used to mutate *Asn*62, *Asn*188, and *Asn*301 to glutamine according to the manufacturer's protocol. The following oligonucleotide primers were used for mutagenesis (with the mutagenic nucleotides in capital letters): *Asn62*, 5′-*gaaagagatatagaaaattcttgCaGgagtcctataaacatgaacagg* ; *Asn188*, 5′-*ctttatattgtgagagtgttcacCaGttcactttaccctcctgggccac* ; and *Asn301*, 5′-*ctttgtggagatgtactatcggCaGgagacgcagcacgagccgtatcc* . Samples were treated with the restriction enzyme DpnI (Stratagene, 10 U) at 37°C for 90 min, and transformed into chemically competent XL10-Gold cells. Plasmids were confirmed by DNA sequencing. Seven mutants were prepared in total: three single mutants [hPAP (N62Q), hPAP (N188Q), and hPAP (N301Q)], three double mutants [hPAP (N62Q, N188Q), hPAP (N62Q, N301Q), and hPAP (N188Q, N301Q)], and one triple mutant [hPAP (N62Q, N188Q, N301Q)].

### Transformation in *P. pastoris*

Each r-hPAP construct was linearized with PmeI, purified using QIAquick PCR Purification Kit (Qiagen) and transformed into electrocompetent *Pichia pastoris* X33 cells according to the EasySelect™ *Pichia* Expression Kit protocol (Invitrogen). The hPAP constructs were stably integrated into the *Pichia* genome at the *AOX1* locus, with transcription driven by the methanol-inducible *AOX1* promoter. To confirm integration, genomic DNA was isolated from Zeocin-resistant colonies by treating with lyticase (25 U in 10 µL water, Sigma-Aldrich) for 10 min at 30°C followed by 10 min at −80°C. Positive transformants with the Mut^+^ phenotype were identified by colony PCR with the 3′ *Pichia* primer (5′-*gcaaatggcattctgacatcc* ) and 5′ *Pichia* primer (5′-*gactggttccaattgacaagc* ) with TITANIUM™ Taq DNA polymerase (Clontech). The predicted molecular weight for each DNA species was as follows: the endogenous *AOX1* alcohol oxidase gene (2.2 kb), hPAP with the α-factor signal peptide (1.65 kb) and hPAP without the signal peptide (1.39 kb).

### r-hPAP protein expression

A single clone from each *P. pastoris* X33 r-hPAP integrant (and an X33 untransformed control) was used to inoculate BMGY media (Invitrogen) at 28°C with shaking at 250 rpm. Cells were grown to a final OD_600_ = 4–5. Cells were harvested by centrifugation at 3,840 rcf for 5 min., and the supernatant was discarded. The cell pellet was resuspended to a final OD_600_ = 1.0 in BMMY media (Invitrogen, 1% methanol). Cells were grown at 28°C with shaking in baffled flasks for 48 h, adding methanol to a final concentration of 1% (v/v) every 24 h. The cells were harvested by centrifugation at 3,840 rcf for 5 min. at 4°C, and the secreted (supernatant) and cellular fractions (cell pellet) were separated. Cells were lysed with acid-washed glass beads (Sigma-Aldrich) according to the EasySelect™ *Pichia* Expression Kit protocol (Invitrogen). Total protein content in each crude supernatant and cell lysate was measured using the Bio-Rad Protein Assay.

### Immunoblotting

Protein samples were analyzed by SDS-PAGE with 4–15% gradient Tris-HCl polyacrylamide gels (BioRad), loading crude secreted protein in the supernatant (0.025 mg/mL) or total protein in the cell lysate (0.25 mg/mL). Proteins were transferred to a nitrocellulose membrane and were probed with primary hPAP antiserum (1∶10,000; Sigma #P5664) followed by anti-rabbit IRDye800 secondary antibody (1∶20,000; Rockland #611-731-127). Blots were imaged at 800 nm using the Licor Odyssey Imaging System.

### Isolation and purification of hPAP

To generate protein for *in vivo* testing, *P. pastoris* X33 were transformed with r-hPAP (200 mL BMGY), were grown to mid-log phase (OD_600_ = 5) and then were induced with 1% methanol in 1 L BMMY media at 28°C for 72 h with shaking. Cells were harvested by centrifugation, and secretory hPAP was precipitated with ammonium sulfate as described [32]. The resulting protein pellet was resuspended in 10 mM sodium acetate, pH 5.3 (15 mL/L original culture volume) and dialyzed against 16 L of 50 mM sodium acetate, pH 5.3. Following dialysis, the crude ammonium sulfate precipitate was concentrated to 2–5 mL total volume in centrifuge filter devices (10,000 MW cutoff; Amicon). The concentrated r-hPAP sample was applied to a HiLoad 26/60 Superdex 200 size-exclusion column (GE Healthcare) at a flow rate of 2 mL/min in 25 mM Tris, pH 7.5 with 150 mM NaCl. Fractions containing r-hPAP were combined (15 mL) and dialyzed against 3 L of 25 mM Bis-Tris, pH 6.5. Following dialysis, r-hPAP was purified to homogeneity by MonoQ anion-exchange chromatography (5/50 GL) in 25 mM Bis-Tris, pH 6.5 (1 mL/min flow rate, elute in 125–150 mM NaCl). The purified recombinant protein was dialyzed in 0.9% saline, pH 5.6. The r-hPAP (N62Q, N188Q) mutant was purified using the same method. All dialysis and purification steps were performed at 4°C.

### Glycosylation assay

Conditioned media containing secreted r-hPAP was concentrated with several rounds of high-speed buffer exchange in 10,000 MW cutoff centrifuge filters (Amicon) with 100 mM sodium acetate, pH 5.3. Total protein concentration was determined following buffer exchange with the BioRad protein assay (BioRad). Reactions (50 µL total volume) containing the concentrated r-hPAP secreted fraction (2 µg total protein) and G7 reaction buffer (50 mM sodium phosphate, pH 7.5, New England Biolabs) were treated with PNGase F (1000 U, New England Biolabs) for 24 h at 37°C. An untreated control (no PNGase F) was incubated at 37°C for 24 h in parallel. Aliquots were removed after 24 h for analysis by SDS-PAGE and the DiFMUP activity assay.

### DiFMUP activity assay

Phosphatase activity of each r-hPAP protein sample was monitored using the EnzChek Phosphatase Assay Kit (Invitrogen). Samples (100 µL total volume) containing 25 µL hPAP protein (0.625 µg crude secreted protein in the supernatant, or 6.25 µg total protein in the crude cell lysate) were diluted with 25 µL of reaction buffer (100 mM sodium acetate, pH 5.3) in a black, clear-bottom 96-well plate (Corning). The reaction was initiated with addition of a fluorogenic substrate, 6,8-difluoro-4-methylumbelliferyl phosphate (DiFMUP, 50 µL, 100 µM final concentration). The fluorescence in each well was recorded (ex: 390 nm, em: 510 nm) every 30 s over a 60 min. total assay time. All samples were assayed in duplicate with and without a known acid phosphatase inhibitor, L-(+)-tartrate (70 mM). Relative activity measurements were determined in the initial (linear) phase of the timecourse. The “endpoint” fluorescence was monitored at 10 min for each reaction, and the activity was calculated with the units RFU/µg protein. Activity for each mutant was also calculated relative to the activity of r-hPAP.

### Behavior

All procedures and behavioral experiments involving vertebrate animals were approved by the Institutional Animal Care and Use Committee at the University of North Carolina at Chapel Hill. All experiments were performed as previously described with male mice during the light phase, raised under a 12∶12 light∶dark cycle [3]. C57BL/6 mice (2–4 months in age) were purchased from Jackson Laboratories, and *A_1_R^−/−^* mice were backcrossed to C57BL/6J mice for 12 generations. All mice were acclimated to the experimenter, the room and the experimental apparatus for 3–5 days prior to behavioral testing. Thermal sensitivity was monitored using the Hargreaves method, where the radiant heat source was calibrated to elicit a paw withdrawal reflex of approximately 10 sec in naïve mice (cutoff time of 20 sec). Mechanical sensitivity was measured with semi-flexible tips attached to an electronic Von Frey apparatus (IITC Life Science). Mice were intrathecally injected (5 µL) with native hPAP (Sigma), r-hPAP, or r-hPAP (N62Q, N188Q) using acute lumbar puncture without anesthesia [33]. Each hPAP protein sample was dialyzed against 0.9% saline, pH 5.6. To maintain antinociceptive activity, we found that it was essential to store this dialyzed hPAP protein at −80°C in single-use aliquots and thaw just prior to injection. The activity of purified r-hPAP protein was characterized with the DiFMUP substrate in comparison to a working stock of 50 U/mL native hPAP. For the purified r-hPAP enzyme, 50 U/mL corresponds to a protein concentration of 0.21 mg/mL. The N-glycosylation mutant, r-hPAP (N62Q, N188Q), was also diluted to 0.21 mg/mL. Complete Freund's adjuvant (20 µL) was injected under the glabrous skin to inflame one hindpaw. Spared nerve injury (SNI) was used to model neuropathic pain [34]. Motor function was measured by the rotarod performance test (cutoff time of 300 sec). Baseline measurements were collected with 3 test iterations, separated by 40 sec for each mouse. Trials were again performed 24 hr following injection of r-hPAP, with 2 iterations separated by 40 sec for each mouse.

## Supporting Information

Figure S1 **r-hPAP clones are stably integrated into the** ***Pichia AOX1*** **locus.** Agarose gel with ethidium bromide staining of r-hPAP integrants into the *AOX1* gene. The number of correctly targeted, Mut^+^ colonies relative to the total number of colonies screened for each clone are shown below each lane.
(TIF)

Figure S2 **L-(+)-tartrate inhibition of r-hPAP mutants.** (A, B) DiFMUP fluorometric enzyme assays of (A) crude cell lysates and (B) crude secreted fractions with L-(+)-tartrate (70 mM). Data are plotted as an average of duplicate trials ± SD.
(TIF)
