# Supplementary figures and images for: Secretion and N-Linked Glycosylation Are Required for Prostatic Acid Phosphatase Catalytic and Antinociceptive Activity
Source: PLoS One. 2012 Feb 28;7(2):e32741. doi: 10.1371/journal.pone.0032741 (PMC3289678; doi:10.1371/journal.pone.0032741)

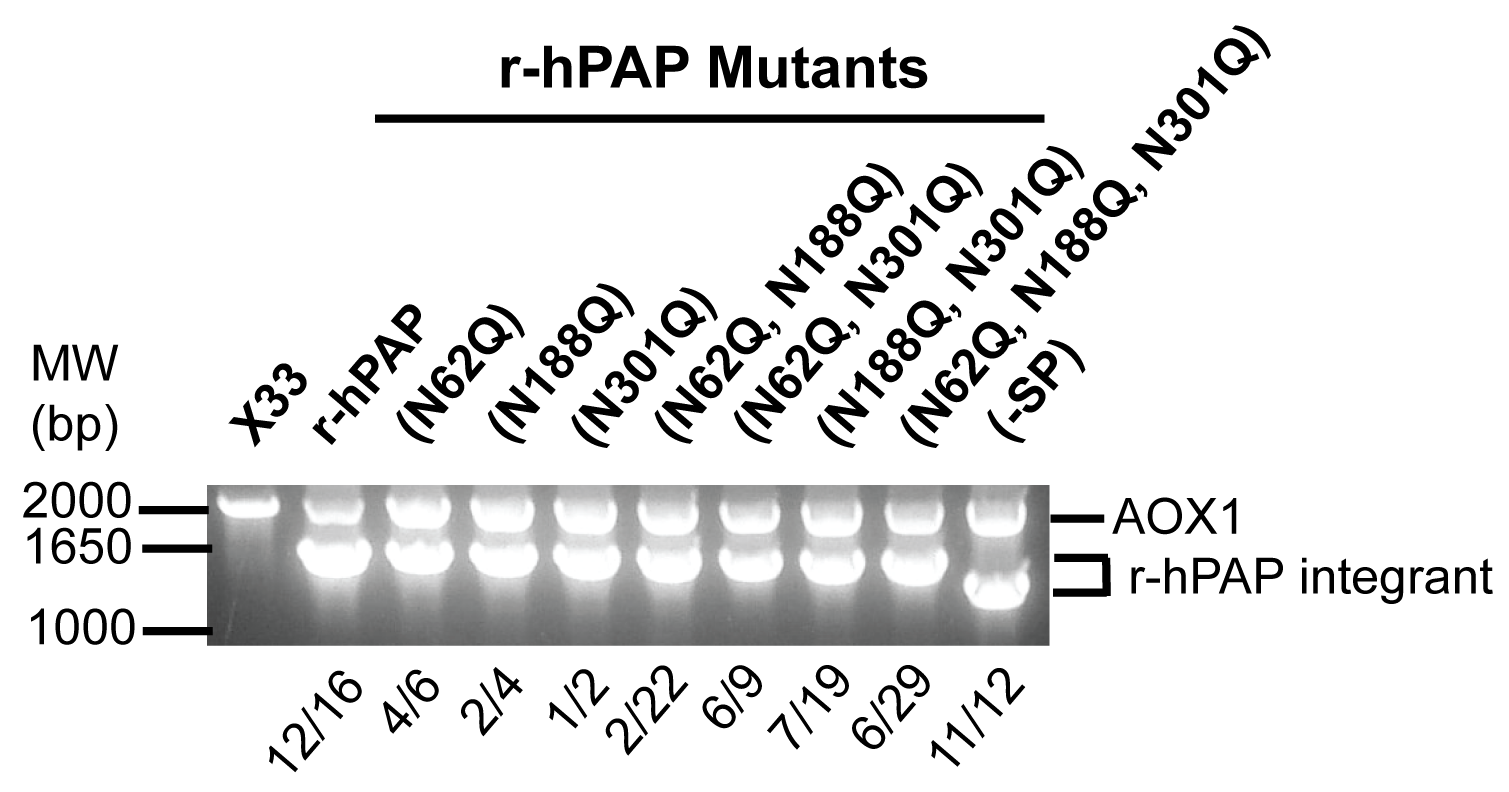

Supplement: Figure S1 — r-hPAP clones are stably integrated into the Pichia AOX1 locus. Agarose gel with ethidium bromide staining of r-hPAP integrants into the AOX1 gene. The number of correctly targeted, Mut+ colonies relative to the total number of colonies screened for each clone are shown below each lane. (TIF) [file pone.0032741.s001.tif]

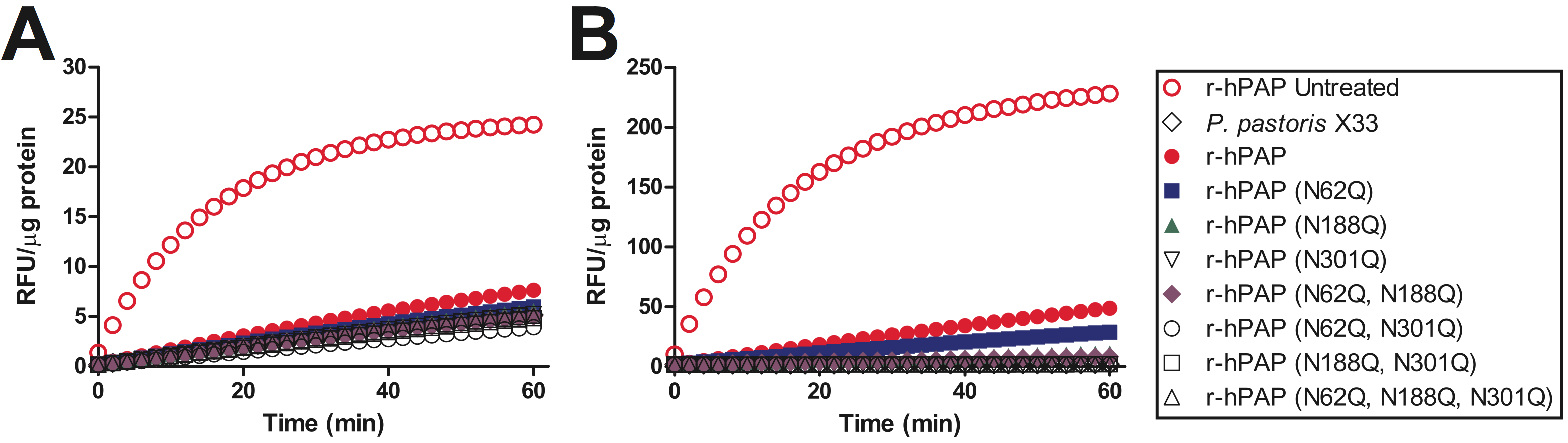

Supplement: Figure S2 — L-(+)-tartrate inhibition of r-hPAP mutants. (A, B) DiFMUP fluorometric enzyme assays of (A) crude cell lysates and (B) crude secreted fractions with L-(+)-tartrate (70 mM). Data are plotted as an average of duplicate trials ± SD. (TIF) [file pone.0032741.s002.tif]
